# Renal NHE3 is required to limit hypokalemia and metabolic acidosis during dietary potassium deficiency

**DOI:** 10.1007/s00424-026-03178-9

**Published:** 2026-06-09

**Authors:** Jennifer Nogueira Coelho, Natalie Hayslip, Rebecca Zoe Halter, Sima Al-Masri, Adriana C. C. Girardi, Maryam Tahmasbi, Jessica A. Dominguez Rieg, Timo Rieg

**Affiliations:** 1 Morsani College of Medicine, Department of Molecular Pharmacology and Physiology, University of South Florida, 12901 Bruce B. Downs Blvd., Tampa, FL 33612 USA; 2 Laboratório de Fisiologia Renal e Cardiometabolismo, Departamento de Cardiopneumologia, Faculdade de Medicina, Universidade de São Paulo, São Paulo, Brazil; 3 James A. Haley Veterans’ Hospital, Tampa, FL USA; 4 Hypertension and Kidney Research Center, University of South Florida, Tampa, FL USA

## Abstract

The sodium-hydrogen exchanger isoform 3 (NHE3) is a critical mediator of proximal tubule and thick ascending limb Na^+^ reabsorption and H^+^/NH_4_^+^ secretion, yet the physiological implications of its upregulation during low K^+^ intake remain poorly understood. We hypothesized that renal NHE3 is important for the acid-base response during K^+^ depletion. To test this, we utilized kidney-specific NHE3 knockout (NHE3^KS−KO^) mice. Following 10 days of a K^+^-deficient diet (< 30 ppm), NHE3^KS−KO^ mice exhibited exacerbated hypokalemia compared to control mice, despite near complete elimination of urinary K^+^ excretion and comparable levels of ROMK and BKα protein abundance in both genotypes. Control and NHE3^KS−KO^ mice developed hypernatremia secondary to a urinary concentrating defect; however, this was associated with a ~ 2-fold greater fluid intake in NHE3^KS−KO^ mice. These effects occurred without changes in glomerular filtration rate. Both genotypes developed metabolic acidosis, which was significantly more severe in NHE3^KS−KO^ mice. This exacerbated acidosis correlated with a ~ 60% lower mRNA expression of glutamine synthetase and a lower urinary NH_4_^+^/creatinine ratio. Furthermore, early histopathological signs of hypokalemic nephropathy were observed exclusively in some of the NHE3^KS−KO^ mice. Our findings demonstrate that renal NHE3 attenuates the severity of hypokalemia and metabolic acidosis during K^+^ restriction, possibly via NH_4_^+^ formation/transport, which might accelerate the development of hypokalemic nephropathy.

**Supplementary Information:**

The online version contains supplementary material available at 10.1007/s00424-026-03178-9.

## Introduction

Challenges to potassium (K^+^) homeostasis can be detrimental to the body since K^+^ is a vital component of many cellular processes, including maintenance of membrane potential, cellular excitability, proliferation, and growth. The kidneys, regulated primarily by aldosterone, play a dominant role in regulating K^+^ homeostasis [44]. Although the distal convoluted tubule, connecting tubule and collecting duct fine-tune K^+^ excretion, ~ 70% of filtered K^+^ is reabsorbed by the proximal tubule [22], as demonstrated by micropuncture studies [6, 36]. Medical textbooks iterate that a positive relationship exists between plasma K^+^ and H^+^ concentrations: hypokalemia leads to metabolic alkalosis; hyperkalemia leads to metabolic acidosis [31]. The proposed mechanism for hypokalemia-induced alkalosis involves increased proximal tubule H^+^ secretion, leading to enhanced HCO_3_^−^ reabsorption [46]. However, the development of metabolic alkalosis during hypokalemia is governed by multiple factors and exhibits significant species variation. While rodents [12, 13, 32, 46] and humans [1, 25, 28] typically develop metabolic alkalosis in response to K^+^ restriction/hypokalemia, dogs [10, 11, 27, 54] and rabbits [38] develop metabolic acidosis under similar conditions. Furthermore, some studies suggest that reductions in GFR, which decrease filtered HCO_3_^-^ load, may be more important than enhanced tubular HCO_3_^−^ reabsorption in generating alkalosis during hypokalemia [15].

The Na^+^/H^+^ exchanger isoform 3 (NHE3), expressed in the proximal tubule and thick ascending limb, was proposed to contribute to H^+^ secretion and subsequently HCO_3_^−^ reabsorption in addition to its role in Na^+^ reabsorption [17]. Early studies using whole body NHE3 knockout (NHE3^−/−^) mice supported this hypothesis [49, 56], as NHE3^−/−^ mice displayed metabolic acidosis consistent with impaired HCO_3_^−^ reabsorption. However, more refined tissue-specific (tubule-specific and proximal tubule specific) knockout models failed to demonstrate significant effects on blood pH or HCO_3_^−^ levels [21, 34, 43, 61], challenging the proposed role of renal NHE3 in acid-base homeostasis. Notably, intestinal epithelial cell-specific NHE3 knockout mice developed severe metabolic acidosis [60], suggesting that the acid-base disturbance in NHE3^−/−^ mice primarily reflects intestinal rather than renal dysfunction. Studies examining renal NHE3 abundance during K^+^ restriction in rodents have yielded variable results, ranging from no change in the renal cortex [41] to ~ 4-fold increases in whole kidney lysates [9] and ~ 7-fold increases in the renal cortex/outer stripe medulla [19]. The greater NHE3 abundance appears consistent with enhanced amiloride-sensitive (unspecific NHE inhibitor) ^22^Na^+^ uptake in brush border membrane vesicles isolated from the renal cortex of K^+^-depleted rats [50] and increased NHE3 mRNA expression in cultured opossum kidney cells exposed to low K^+^ medium [3].

Given the variable effects of hypokalemia on acid-base status and NHE3 abundance reported in the literature, we hypothesized that mice lacking renal NHE3 would develop severe acid-base disturbances in response to K^+^-deficient diet. Our major finding was that, compared to control mice, NHE3^KS−KO^ mice developed more pronounced hypokalemia and greater metabolic acidosis without changes in GFR. These results indicate that renal NHE3 is required to mitigate the severity of both hypokalemia and metabolic acidosis during K^+^ depletion as well as hypokalemic nephropathy. Additionally, we found that renal NHE3 is necessary for increasing urinary Na^+^ excretion in response to hypokalemia. Further studies are needed to identify the factors that determine whether metabolic acidosis or alkalosis develops during hypokalemia in different experimental models and clinical settings.

## Materials and methods

### Animals

All animal experiments were conducted at the University of South Florida in accordance with the Guide for the Care and Use of Laboratory Animals (National Institutes of Health, Bethesda, MD, USA) and were approved by the Institutional Animal Care and Use Committee (R11592). The generation and phenotype of tubule-specific NHE3 knockout mice have been described previously [20, 45]. Mice were housed under a 12:12-hour light-dark cycle in isolated ventilated cages with free access to control diet (1% K^+^, TD.88238, Envigo, Madison, WI) and tap water. Age-matched, 3- to 6-month-old male and female mice (control, NHE3^loxlox^) or tubule-specific NHE3 knockout mice (NHE3^KS−KO^) were used for experiments. To knockout NHE3 along the renal tubule, a Pax8^Cre^ driver was used [8, 45].

### Experimental protocols

Mice were maintained on control diet (1% K^+^) during an initial baseline period. During this baseline period, blood (collected via brief isoflurane anesthesia) and spontaneous voided urine samples were collected and GFR was measured (see “GFR measurement” section). Following baseline assessments, control and NHE3^KS−KO^ mice were switched to a K^+^-deficient diet (15–30 ppm K^+^, TD.88239, Envigo) for 10 days. Body weight, water intake, and food intake were monitored daily through the study period in home cages where mice were pair-housed. Urine samples were collected by reflex urination. On day 9, GFR measurements were repeated. On day 10, mice were anesthetized with isoflurane, and a blood sample was collected from the retrobulbar plexus. Under continued isoflurane anesthesia, the left kidney was removed, weighed and cut in half. One half was immediately snap-frozen and stored for subsequent immunoblot analysis (see “Immunoblot analysis” section). The other half was cut into small pieces, and stabilized in RNAlater solution (Thermo Fisher Scientific, Middletown, VA) for gene expression analysis (see “Quantitative real-time polymerase chain reaction” section). Mice were subsequently perfused through the left ventricle with 4% paraformaldehyde (PFA) in phosphate buffered saline (PBS) and the kidney was removed. Afterward, tissue was fixed overnight in 4% PFA in PBS and prepared for histopathology (see “Histopathology” section).

### GFR measurements

GFR was measured using plasma kinetics of FITC-Sinistrin (1% solution, 2 µl*g^− 1^ body weight, Fresenius Kabi, Linz, Austria) by using transdermal miniaturized fluorescence detectors (MediBeacon, Mannheim, Germany). In brief, after hair removal, the detectors were attached to the skin of the mouse, followed by retro-orbital injection of FITC-Sinistrin under brief isoflurane anesthesia. Changes of FITC-Sinistrin intensity were recorded for 90 min. After the experiment was finished, the fluorescence detectors were removed, and the data were downloaded and analyzed using MediBeacon Studio 3 software (3.1.2 Rev: 128).

### Analysis of blood and urine samples

Blood chemistry from baseline and terminal blood was determined by an OPTI^®^ CCA-TS2 blood gas analyzer using an E-Cl^−^ Type cassette (OPTIMedical, Roswell, GA). Plasma aldosterone was determined by enzyme linked immunosorbent assay (ADI-900-173, Enzo Life Sciences, Inc. Farmingdale, NY). Urinary Cl^−^ was analyzed by the thiocyanide method (Stanbio Laboratory, Boerne, TX), urinary Na^+^ and K^+^ were measured by flame photometry (BWB Technologies Ltd., Berkshire, UK), urinary NH_4_^+^ was measured by a QuantiFluo™ ammonia assay kit (BioAssay Systems, Hayward, CA), and urinary creatinine was determined by the Jaffe reaction (Enzo Biochem Inc., Farmingdale, NY). Urine osmolality was measured using an Osmomat 3000 (Gonotec, Berlin, Germany) and urine pH was determined using a pH electrode immediately after collection (9810BN, Thermo Fisher Scientific, Pittsburgh, PA).

### Immunoblot analysis

Kidney tissue was homogenized in dissection buffer (250 mmol*L^− 1^ sucrose, 10 mmol*L^− 1^ triethanolamine buffer) containing protease inhibitor cocktail (Thermo Fisher Scientific) and Halt^®^ phosphatase inhibitor cocktail (Thermo Fisher Scientific). Homogenates were centrifuged at 1,000 x g for 10 min. Equal lane loading (20 µg) was achieved using a Bio-Rad DC Protein assay (Bio-Rad Laboratories, Richmond, CA). Samples were resolved on NuPAGE 4–12% Bis-Tris gels in 3-(N-morpholino)propanesulfonic acid buffer. Gel proteins were transferred to polyvinylidene fluoride membranes and immunoblotted with antibodies against NHE3 (rabbit, 1:500, AB3085, Millipore, Billerica, MA), ROMK (rabbit, 1:800, APC-001, Alomone Labs, Jerusalem, Israel) BKα subunit (rabbit, 1:1000, APC-021, Alomone Labs) and β-actin (rabbit, 1:5000, Sigma-Aldrich). The specificity of the NHE3, ROMK and BKα subunit antibodies has been confirmed previously in the respective knockout mice [35, 61, 62]. Detection was performed with secondary antibodies against rabbit (IRDye^®^ 800CW donkey anti-rabbit IgG, 1:5000) or mouse (IRDye^®^ 680RD donkey anti-mouse IgG, 1:5000) and detected with an Odyssey^®^ CLx (LI-COR Biosciences, Lincoln, NE). Densitometric analysis was performed by using Image Studio Lite Version 6.0 (LI-COR Biosciences).

### Quantitative real-time-polymerase chain reaction

Kidney stored in RNAlater was processed for mRNA analysis as described previously [52, 61]. Tissue was homogenized using a Tissue Tearor (Bartlesville, OK) and QIAshredder (Qiagen, Valencia, CA) according to the manufacturer’s instructions. Total RNA was purified using the RNeasy Plus Mini Kit (Qiagen). Complementary DNA was made by reverse transcribing total RNA using a QuantiTect reverse transcription kit (Qiagen) according to the manufacturer’s instructions. Quantitative RT-PCR was performed using TaqMan^Tm^ Gene Expression Master Mix (Fisher Scientific) in a QuantStudio™ 12K Flex PCR System (Applied Biosystems, Foster City, CA). The template concentration was 50 ng cDNA per 20 µL reaction (performed in triplicate) and was used in conjunction with primer pairs specific for phosphoenolpyruvate carboxykinase (Pepck, Mm01247058_m1), glutaminase (Gls, Mm01257297_m1), glutamine synthetase (Glul, Mm00725701_s1) and rhesus blood group C glycoprotein (Rhcg, Mm00451199_m1) with glyceraldehyde-3-phosphate dehydrogenase (Gapdh, Mm99999915_g1) as a reference gene. Data analysis used the 2^−ΔΔ^Ct method (where Ct is threshold cycle) and normalized to GAPDH expression and compared with control [61].

### Histopathology

PFA-fixed kidneys were paraffin embedded and sectioned at 4–6 μm. After deparaffinization and rehydration, sections were stained with hematoxylin and eosin (H&E) and Masson Trichrome (Reliance Pathology Partners, Tampa, FL). Tubulointerstitial injury in stained kidney sections was graded by using the following scheme: Grade 1 (< 10%), grade 2 (10–25%), grade 3 (25–50%), grade 4, (50–75%); and grade 5, (75–100%). The highest score seen in sections was reported for each mouse. All scoring and histopathological assessment was performed by a pathologist (M.T.) blinded to sample identity.

### Fluorescent staining

Staining procedures were described previously in detail [18, 47, 61]. Goat anti-rabbit Texas Red™ (10 µg*mL^− 1^; TI-1000, Vector Laboratories, Newark, CA) was used for visualization of ROMK labeling (2 µg*mL^− 1^, APC-001, Alomone Labs). The slides were mounted with VECTASHIELD^®^ Antifade Mounting Medium with DAPI (H-1200, Vector Laboratories). An Olympus (Center Valley, PA) FV3000 Multiphoton Excitation Laser Scanning Microscope with an UPlanFL N 60x/0.65–1.25 oil objective was used for imaging of labeled sections. ImageJ Version 1.54p (National Institutes of Health, MD) was used for quantitative analysis performed by an investigator (J.A.D.R.) blinded to sample identity. Identical acquisition and analysis settings were used across all samples. For each animal, ten regions of interest (ROIs) were manually defined within the renal cortex, avoiding saturated pixels and obvious artifacts. Background fluorescence was measured from an adjacent region lacking specific signal. For each ROI, the integrated density (IntDen) and area were recorded, and the mean background fluorescence was calculated from the background ROI. Fluorescence intensity was expressed as corrected total cell fluorescence (CTCF) using the formula: CTCF=IntDen-(Area×Mean background). The average CTCF from the ten ROIs was calculated to yield a single value per animal.

### Data analysis and statistics

Data are expressed as mean ± SEM. Unpaired Student’s *t*-test was performed to analyze statistical differences between two groups. Two-way ANOVA followed by two-stage linear step-up procedure of Benjamini, Krieger and Yekutieli or Šidák’s multiple comparison test, as indicated in figure legends, were used to test for significant differences between genotype or experimental conditions. All data were analyzed via GraphPad Prism Version 10.6.1 (Boston, MA) or SigmaPlot Version 14.5 (San Jose, CA). Significance was considered at *P* < 0.05.

## Results

### Dietary K^+^ restriction causes polyuria and polydipsia

**Fig. 1:**
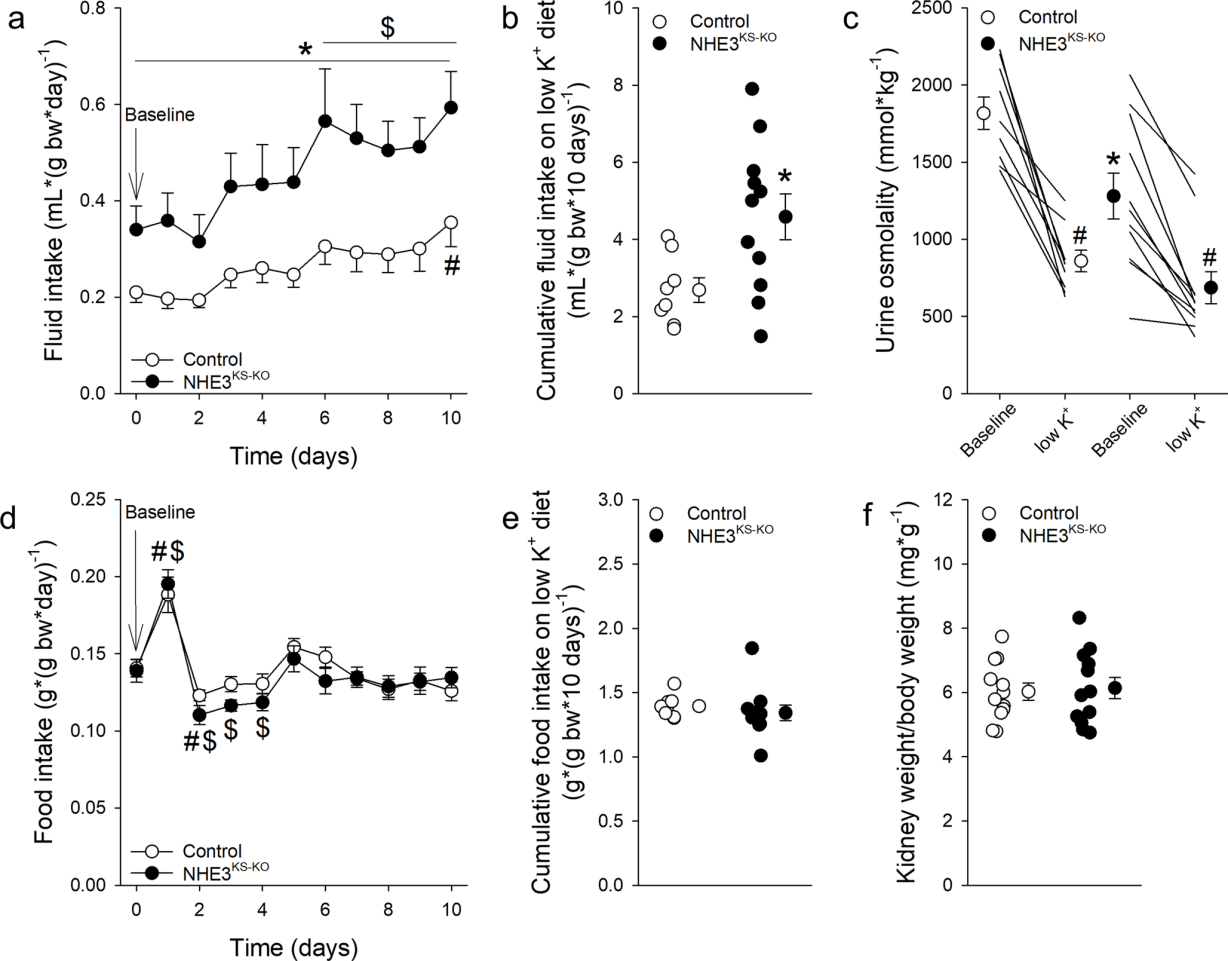
Baseline physiological parameters and responses to dietary K^+^ deficiency. Under baseline conditions, fluid intake (**A**) was significantly greater in NHE3^KS−KO^ compared to control mice, consistent with their urinary concentrating defect (**C**). Following K^+^-deficient diet, fluid intake (**A**) increased in both genotypes and remained significantly greater in NHE3^KS−KO^ mice, as reflected in cumulative fluid intake (**B**). (**C**) K^+^-deficient diet significantly lowered urine osmolality in both genotypes. (**D**) Food intake followed a similar temporal pattern in both genotypes, resulting in comparable cumulative food intake between groups (**E**). Left kidney-to-body weight ratios were not significantly different between genotypes at the end of the experimental period (**F**). Single data points with connecting lines (showing paired baseline and low K⁺ values for each animal) and/or mean ± SEM are shown. Data were analyzed by a two-way mixed effects model followed by two-stage linear step-up procedure of Benjamini, Krieger and Yekutieli (A, D), 2-way ANOVA followed by Šídák’s multiple comparisons test (**C**), and an unpaired Student’s t-test (**B**, **E**). *P < 0.05 versus control mice, ^#^*P* < 0.05 versus baseline for control mice, ^$^*P* < 0.05 versus baseline for NHE3^KS−KO^ mice. For control *n* = 5 ♂ and 7 ♀ mice; for NHE3^KS−KO^ *n* = 7 ♂ and 5 ♀ mice

Under baseline conditions, NHE3^KS−KO^ mice show significantly greater fluid intake compared to control mice, consistent with their significantly lower urine osmolality (Fig. 1A, C), as previously described [21]. No significant differences in food intake were observed under baseline conditions (Fig. 1D). Following the switch to K^+^-deficient diet, fluid intake significantly increased in both genotypes (Fig. 1A), consistent with the development of a urinary concentrating defect (Fig. 1C). Cumulative fluid intake over the 10-day experimental period was significantly greater in NHE3^KS−KO^ compared to control mice (Fig. 1B). Food intake remained similar between genotypes throughout the experimental period (Fig. 1D), as evidenced by comparable cumulative food intake (Fig. 1E). Both genotypes showed similar feeding patterns after the switch to K^+^-deficient diet (Fig. 1D). At the end of the experimental period no significant differences in kidney-to-body weight ratios were observed between genotypes (Fig. 1E).

### Renal NHE3 attenuates hypokalemia in response to dietary K^+^ restriction

**Fig. 2:**
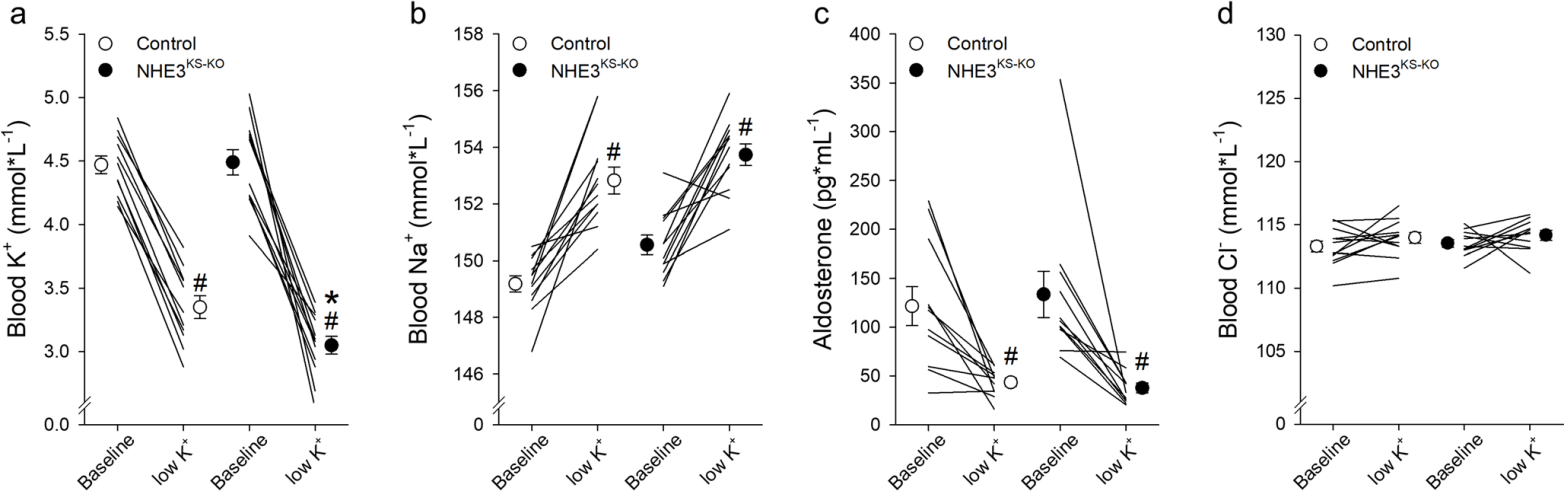
Blood responses to dietary K^+^ deficiency in control and NHE3^KS−KO^ mice. Physiological parameters in control and NHE3^KS−KO^ mice at baseline and after 10 days of K^+^-deficient diet. (**A**) K^+^-deficient diet significantly decreased blood K^+^ in both genotypes, with a greater decline in NHE3^KS−KO^ mice compared to control mice. (**B**) Both genotypes developed hypernatremia to a similar extent. (**C**) Plasma aldosterone was reduced similarly in both genotypes. (**D**) Blood Cl^−^ remained unchanged in both genotypes. Single data points with connecting lines (showing paired baseline and low K⁺ values for each animal) and mean ± SEM are shown. Data were analyzed by a 2-way ANOVA followed by Šídák’s multiple comparisons test. *P < 0.05 versus control mice, ^#^*P* < 0.05 versus baseline same genotype. For control *n* = 5 ♂ and 7 ♀ mice; for NHE3^KS−KO^ *n* = 7 ♂ and 5 ♀ mice

Under baseline conditions, blood Na^+^, K^+^, Cl^−^, and aldosterone levels did not significantly differ between genotypes (Fig. 2A-D). Following the switch to K^+^-deficient diet, blood K^+^ significantly decreased in both genotypes (Fig. 2A). However, the magnitude of decline was significantly greater in NHE3^KS−KO^ mice compared to control mice (-1.44 ± 0.15 versus -1.12 ± 0.05 mmol*L^− 1^, *P* < 0.05), indicating more severe hypokalemia in the absence of renal NHE3. Both genotypes developed hypernatremia to a similar extent (Fig. 2B). Plasma aldosterone significantly decreased to a similar extent in both genotypes (Fig. 2C), as expected during K^+^ depletion. Blood Cl^−^ remained unchanged in both genotypes following K^+^-deficient diet (Fig. 2D).

### Natriuresis in response to dietary K^+^ restriction depends on renal NHE3

**Fig. 3:**
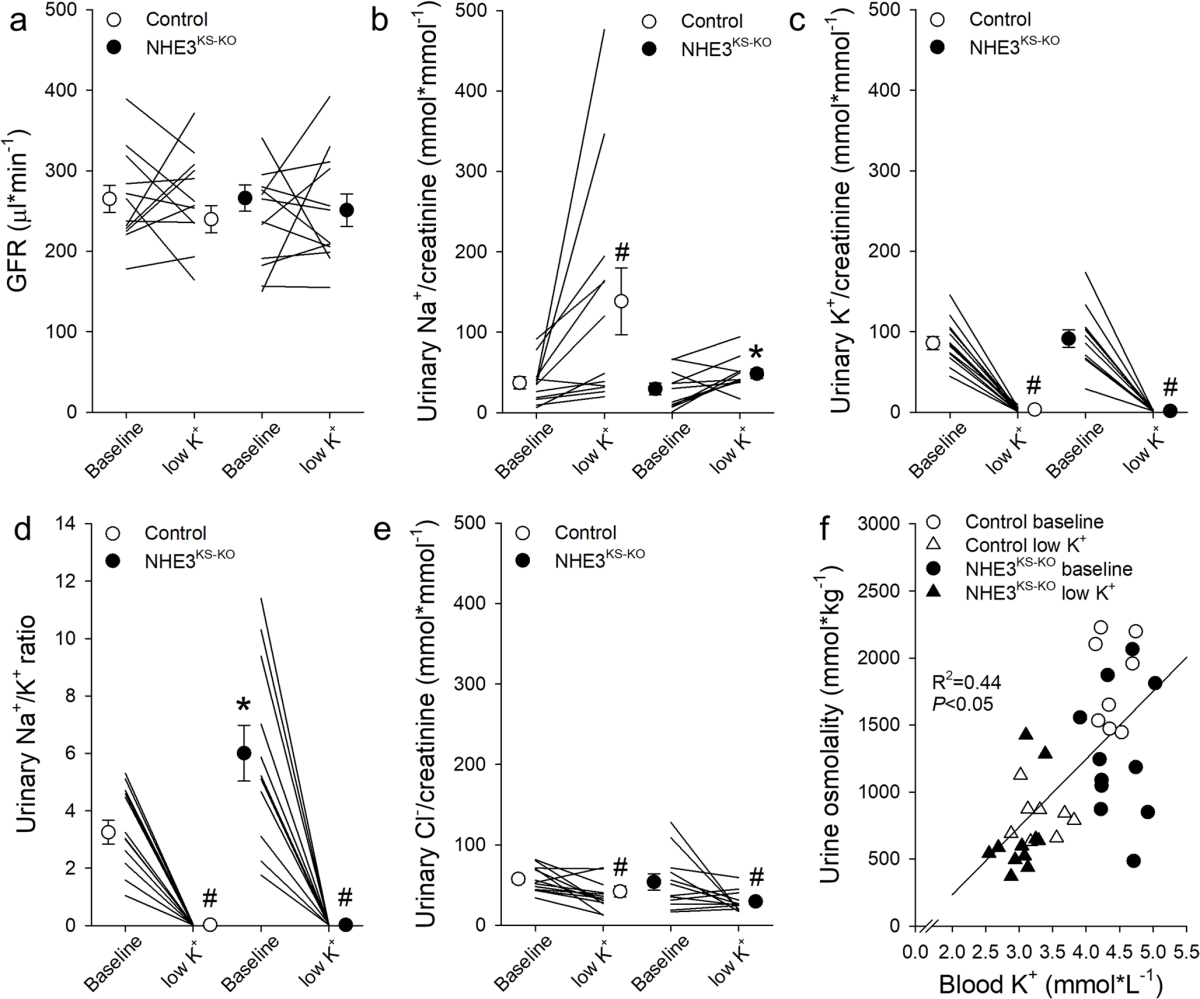
Kidney and urine responses to dietary K^+^ deficiency in control and NHE3^KS−KO^ mice. GFR and urinary electrolyte excretion in control and NHE3^KS−KO^ mice at baseline and after 10 days of K^+^-deficient diet. (**A**) GFR was significantly lower under baseline conditions in NHE3^KS−KO^ mice compared to control mice, but K^+^-deficient diet did not alter GFR in either genotype. (**B**) Urinary Na^+^/creatinine was significantly increased in control mice but remained unchanged in NHE3^KS−KO^ mice. (**C**) Urinary K^+^/creatinine, (**D**) urinary K^+^/Na^+^ ratio, and (**E**) urinary Cl^-^/creatinine decreased similarly in both genotypes. (**F**) A positive correlation was observed between blood K^+^ concentration and urine osmolality. Single data points with connecting lines (showing paired baseline and low K⁺ values for each animal) and mean ± SEM are shown. Data were analyzed by a 2-way ANOVA followed by Šídák’s multiple comparisons test. *P < 0.05 versus control mice, ^#^*P* < 0.05 versus baseline same genotype. For control *n* = 5 ♂ and 7 ♀ mice; for NHE3^KS−KO^ *n* = 7 ♂ and 5 ♀ mice

To exclude the possibility that changes in GFR contributed to alterations in blood and urinary electrolytes or acid-base parameters, we measured GFR at baseline and after 9 days of K^+^-deficient diet. Under baseline conditions, GFR was slightly but significantly lower in NHE3^KS−KO^ mice compared to control mice (Fig. 3A), as described previously [21]. K^+^-deficient diet did not significantly alter GFR in either genotype. Urinary Na^+^/creatinine ratios were not significantly different between genotypes under baseline conditions. Following the switch to K^+^-deficient diet, urinary Na^+^/creatinine ratios significantly increased in control mice but remained unchanged in NHE3^KS−KO^ mice (Fig. 3B), indicating that renal NHE3 is required for the natriuretic response to K^+^ depletion. Urinary K^+^/creatinine ratios were not significantly different between genotypes under baseline conditions. Both genotypes exhibited a similar and significant decrease in urinary K^+^/creatinine ratios in response to K^+^-deficient diet (Fig. 3C), with K^+^ nearly completely eliminated from the urine. The urinary K^+^/Na^+^ ratio was ~ 3.3 in control mice under baseline conditions (Fig. 3D), consistent with the dietary composition of 1% K^+^ and 0.3% Na^+^. NHE3^KS−KO^ mice exhibited a significantly greater urinary K^+^/Na^+^ ratio under baseline conditions compared to control mice. Following K^+^ restriction, this ratio was significantly reduced to a similar extent in both genotypes due to near-complete elimination of urinary K^+^. Urinary Cl^−^/creatinine ratios were not significantly different between genotypes under baseline conditions. Both genotypes showed a comparable and significant decrease in urinary Cl^−^/creatinine ratios following K^+^-deficient diet (Fig. 3E). Consistent with the development of nephrogenic diabetes insipidus and polydipsia, a positive correlation was observed between blood K^+^ concentration and urine osmolality (Fig. 3F).

### Renal NHE3 protects against dietary K^**+**^ restriction-induced metabolic acidosis and is required for adaptive urine alkalinization

**Fig. 4:**
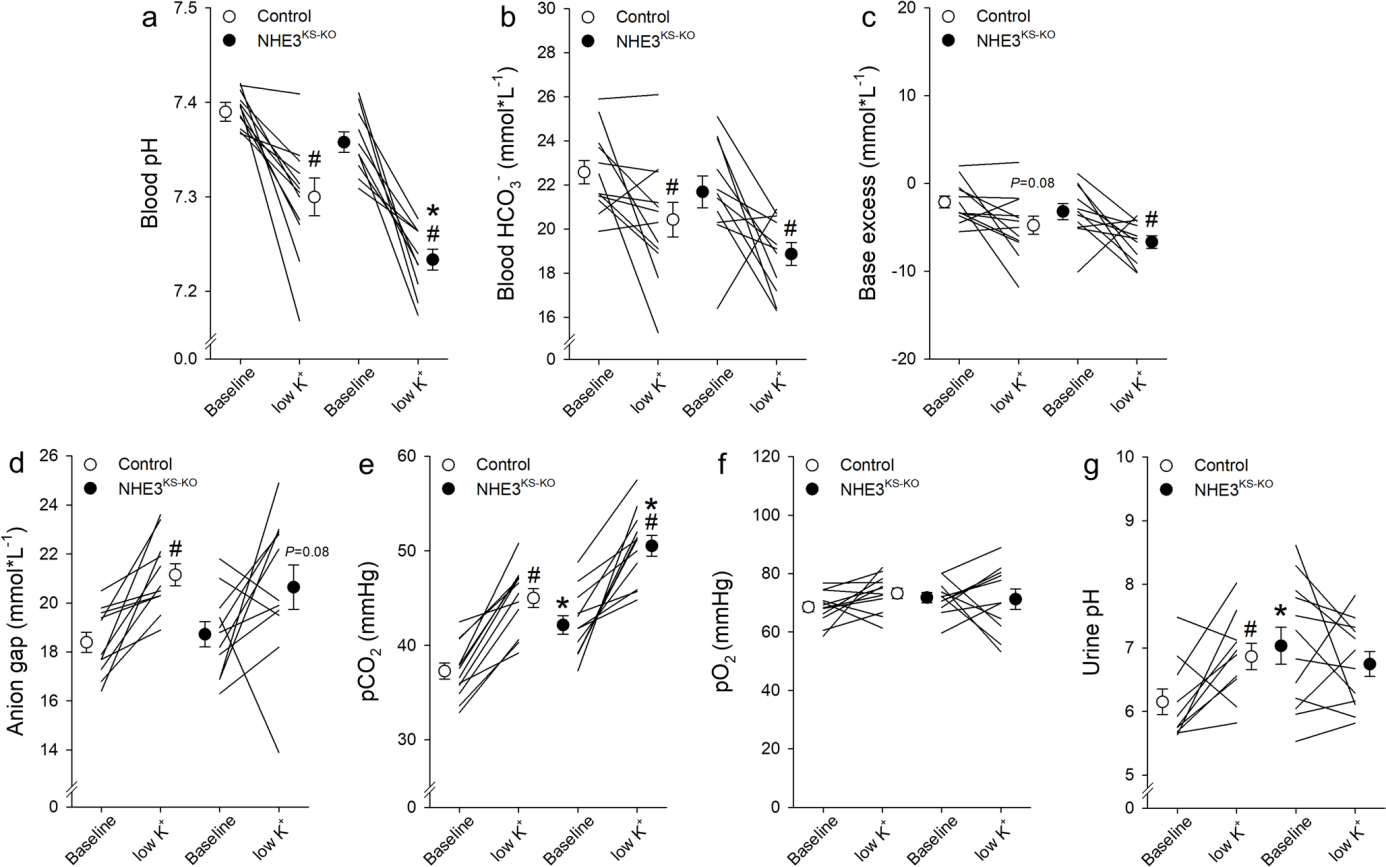
Acid-base parameters in responses to dietary K^+^ deficiency in control and NHE3^KS−KO^ mice. Acid-base parameters in blood and urine from control and NHE3^KS−KO^ mice at baseline and after 10 days of K^+^-deficient diet. Contrary to the expected development of metabolic alkalosis, both genotypes developed metabolic acidosis in response to K^+^ depletion. (**A**) Blood pH decreased significantly in both genotypes, with a more pronounced decrease in NHE3^KS−KO^ mice compared to control mice. (**B**) Blood HCO_3_^−^ concentration decreased similarly in both genotypes. (**C**) Base excess decreased significantly in NHE3^KS−KO^ mice but did not reach statistical significance in control mice (*P* = 0.08). (**D**) Anion gap increased significantly in control mice but showed only a trend toward increase in NHE3^KS−KO^ mice (*P* = 0.08). (**E**) Partial pressure of CO₂ increased significantly in both genotypes, with a greater increase in NHE3^KS−KO^ mice compared to control mice. (**F**) Partial pressure of O₂ was not significantly different between genotypes nor significantly affected by K^+^ depletion. (**G**) Urine pH increased significantly in control mice but remained unchanged in NHE3^KS−KO^ mice. Single data points with connecting lines (showing paired baseline and low K⁺ values for each animal) and mean ± SEM are shown. Data were analyzed by a 2-way ANOVA followed by Šídák’s multiple comparisons test. *P < 0.05 versus control mice, ^#^*P* < 0.05 versus baseline same genotype. For control *n* = 5 ♂ and 7 ♀ mice; for NHE3^KS−KO^ *n* = 7 ♂ and 5 ♀ mice

Since alterations in K^+^ homeostasis are associated with changes in acid-base homeostasis, we measured various blood and urinary acid-base parameters. At baseline, blood pH, blood HCO_3_^−^, base excess and anion gap were not significantly different between genotypes (Fig. 4A-D). Partial pressure of CO_2_ was significantly greater in NHE3^KS−KO^ mice under baseline conditions (Fig. 4E). Partial pressure of O₂ was not significantly different between genotypes under baseline conditions (Fig. 4F). Consistent with previous reports [21], NHE3^KS−KO^ mice exhibited more alkaline urine under baseline conditions compared to control mice (Fig. 4G). Following K^+^-deficient diet, blood pH significantly decreased in both genotypes; however, the decline was significantly greater in NHE3^KS−KO^ mice compared to control mice (Fig. 4A), indicating more severe metabolic acidosis in the absence of renal NHE3. Blood HCO_3_^−^ significantly decreased to a similar extent in both genotypes (Fig. 4B). Base excess significantly decreased in NHE3^KS−KO^ mice but did not reach statistical significance in control mice (Fig. 4C). Partial pressure of CO_2_ significantly increased in both genotypes, with a greater increase in NHE3^KS−KO^ mice compared to control mice (Fig. 4E). No effect on partial pressure of O_2_ was observed in either genotype (Fig. 4F). Urine pH significantly increased in control mice but remained unchanged in NHE3^KS−KO^ mice (Fig. 4G), suggesting either impaired renal acid excretion in the absence of NHE3 or that maximum urine alkalinization had been reached.

### Protein abundance of ROMK and BKα subunit are not significantly different in response to K^**+**^ deficient diet

**Fig. 5:**
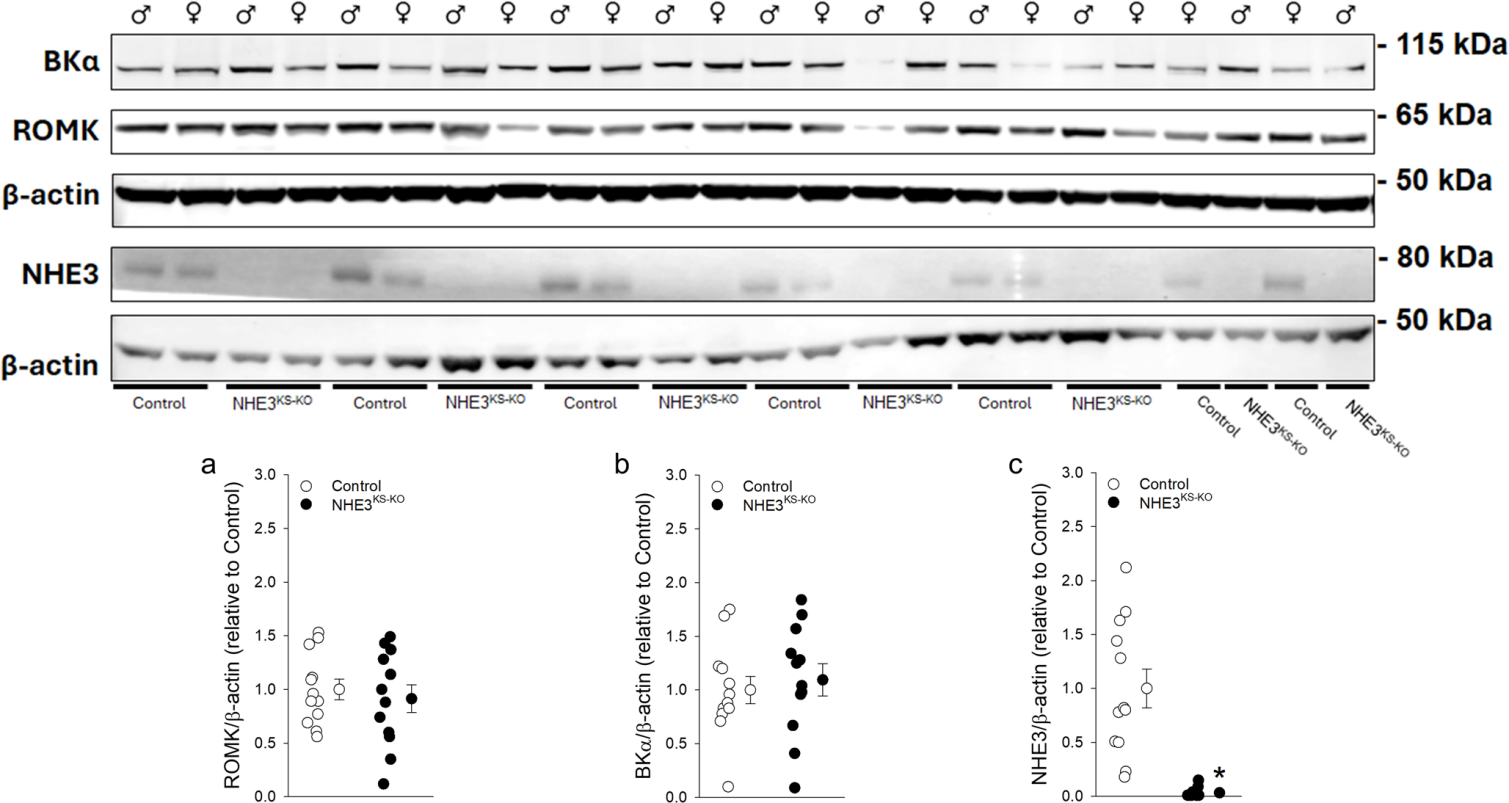
Protein abundance of ROMK, BKα subunit and NHE3 in responses to dietary K^+^ deficiency in control and NHE3^KS−KO^ mice. Protein abundance in whole kidney lysates from control and NHE3^KS−KO^ mice after 10 days of K^+^-deficient diet. (**A**) ROMK and (**B**) BKα subunit expression were not significantly different between genotypes. (**C**) NHE3 protein was completely absent in NHE3^KS−KO^ mice, confirming successful kidney-specific deletion of NHE3. Single data and mean ± SEM are shown. Data were analyzed by an unpaired Student’s *t*-test. *P < 0.05 versus control mice. For control *n* = 5 ♂ and 7 ♀ mice; for NHE3^KS−KO^ *n* = 7 ♂ and 5 ♀ mice

Basal K^+^ secretion in the aldosterone-sensitive distal nephron is mediated primarily by ROMK channels, whereas BK channels contribute significantly to renal K^+^ secretion in response to increased tubular flow rate [5, 47]. To determine whether differences in K^+^ channel expression contributed to the more severe hypokalemia observed in NHE3^KS−KO^ mice, we measured ROMK and BK channel protein abundance at the end of the experimental period. Despite the significantly lower plasma K^+^ concentrations in NHE3^KS−KO^ compared to control mice, no significant differences were observed between genotypes in the protein abundances of ROMK (Fig. 5A) and BKα subunit (Fig. 5B). As expected, renal NHE3 protein abundance was completely absent in NHE3^KS−KO^ mice (Fig. 5C), confirming functionality of the Cre recombinase-loxP system.

### Similar ROMK labeling in response to dietary K^+^ restriction in control and NHE3^KS−KO^ mice

**Fig. 6:**
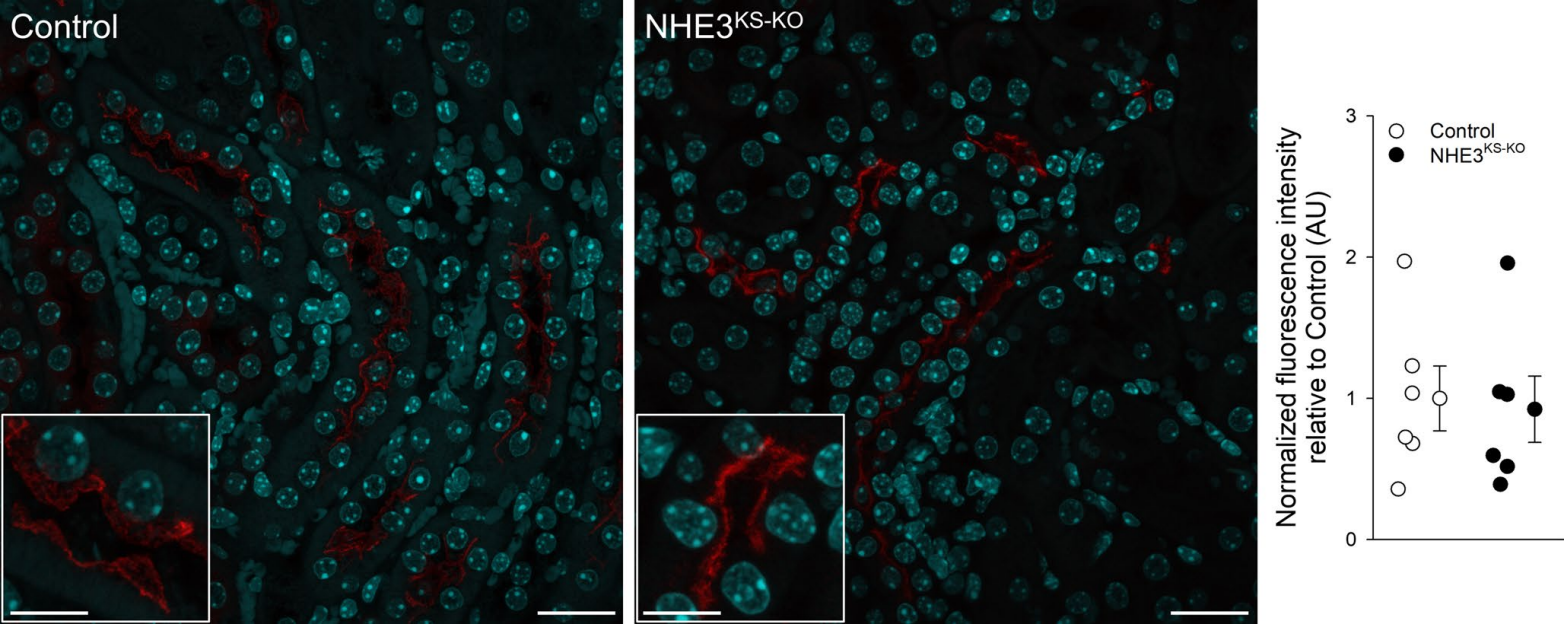
Effect of dietary K^+^ restriction on ROMK localization and abundance. Representative confocal microscopy images of renal cortical ROMK expression in kidney sections from control and NHE3^KS−KO^ mice after 10 days of dietary K^+^ restriction. ROMK localization and ROMK fluorescent intensity were not significantly different between genotypes. Data were analyzed by an unpaired Student’s *t*-test. For control *n* = 3 ♂ and 3 ♀ mice; for NHE3^KS−KO^ *n* = 3 ♂ and 3 ♀ mice. Scale bar of 50 μm and 25 μm (insets) are shown

Since Western blotting does not allow for localization of proteins, we studied renal ROMK localization. Connecting tubules were identified by their histotopography (exclusive located in the renal cortex) and by the presence of ROMK-negative intercalated cells in the epithelial lining. After 10 days of K^+^-deficient diet, no differences in apical localization of ROMK between genotypes were observed. Quantification of ROMK labeling intensity confirmed no significant differences between genotypes (Fig. 6).

### Lack of renal NHE3 is associated with reduced glutamine synthetase mRNA expression in response to dietary K^**+**^ restriction

**Fig. 7:**
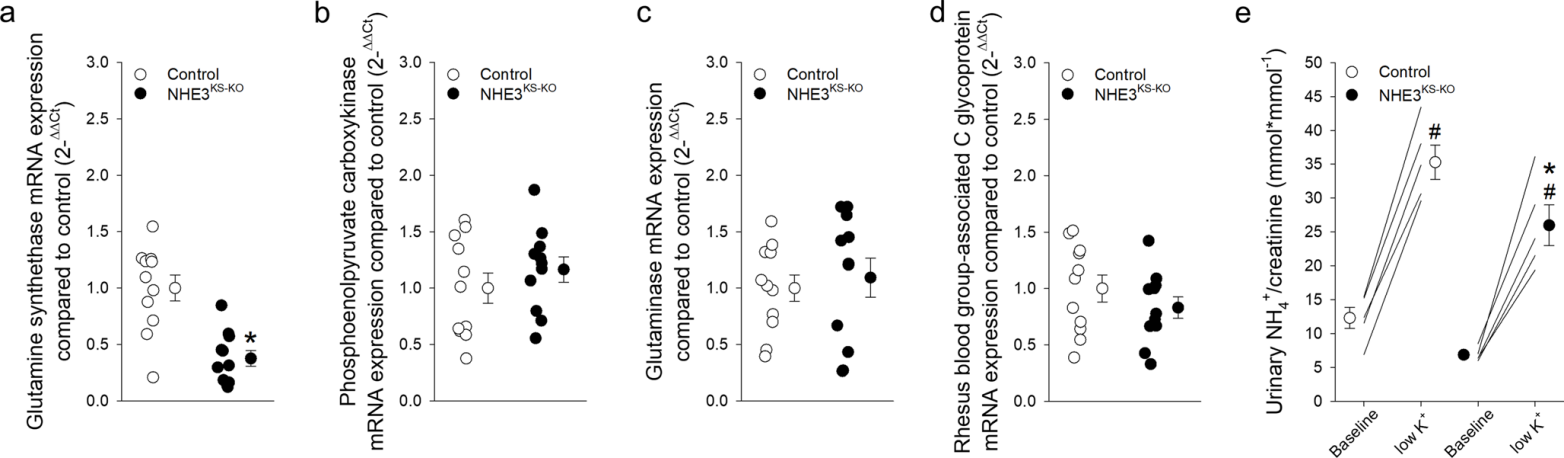
Reduced urinary NH_4_^+^ and glutamine synthetase messenger RNA expression in NHE3^KS−KO^ mice in response to K^+^ deficient diet. Analysis of mRNA expression of NH_4_^+^ metabolism and transporting genes in whole kidney from control and NHE3^KS−KO^ mice after 10 days of K^+^-deficient diet. (**A**) Glutamine synthetase (Glul) mRNA expression was significantly lower in NHE3^KS−KO^ mice compared to control mice. No significant differences were detected between genotypes in the expression of (**B**) phosphoenolpyruvate carboxykinase (Pepck), (**C**) glutaminase (Gls), or (**D**) rhesus blood group C glycoprotein (Rhcg). (**E**) Urinary NH_4_^+^/creatinine significantly increased in both and control and NHE3^KS−KO^ mice but remained significantly lower in response to dietary K^+^ deficiency in NHE3^KS−KO^ mice. Single data and mean ± SEM are shown. For A-D, data were analyzed by an unpaired Student’s *t*-test. *P < 0.05 versus control mice. For control *n* = 4 ♂ and 7 ♀ mice; for NHE3^KS−KO^ *n* = 6 ♂ and 5 ♀ mice. For E, data were analyzed by a 2-way ANOVA followed by Šídák’s multiple comparisons test. *P < 0.05 versus control mice, ^#^*P* < 0.05 versus baseline same genotype. For control *n* = 5 ♀ mice; for NHE3^KS−KO^ *n* = 5 ♀ mice

NH_4_^+^ and K^+^ homeostasis are closely interconnected. Changes in total body K^+^ directly regulates renal ammonia production, and NH_4_^+^, in turn, influences K^+^ excretion and acid-base balance. At the end of the experimental period, mRNA expression of glutamine synthetase (Glul), an enzyme that converts glutamate and NH_4_^+^ into glutamine, was significantly (~ 60%) lower in NHE3^KS−KO^ mice compared to control mice (Fig. 7A). This reduction in Glul expression is consistent with decreased NH_4_^+^ recycling and may contribute to the more severe metabolic acidosis observed in NHE3^KS−KO^ mice. Messenger RNA expression of phosphoenolpyruvate carboxykinase (Pepck), glutaminase (Gls), and Rh family C glycoprotein (Rhcg) did not differ between genotypes (Fig. 7B-D), suggesting that the differential acid-base response was not mediated by changes in these key ammoniagenic or NH_4_^+^ transport proteins. Functionally, urinary NH_4_^+^/creatinine ratios were not significantly different under baseline conditions between genotypes; however, in response to K^+^-deficient diet, the increase in urinary NH_4_^+^/creatinine ratios was significantly smaller in NHE3^KS−KO^ compared to control mice (Fig. 7E).

### NHE3^KS−KO^ mice show early signs of hypokalemic nephropathy

**Fig. 8:**
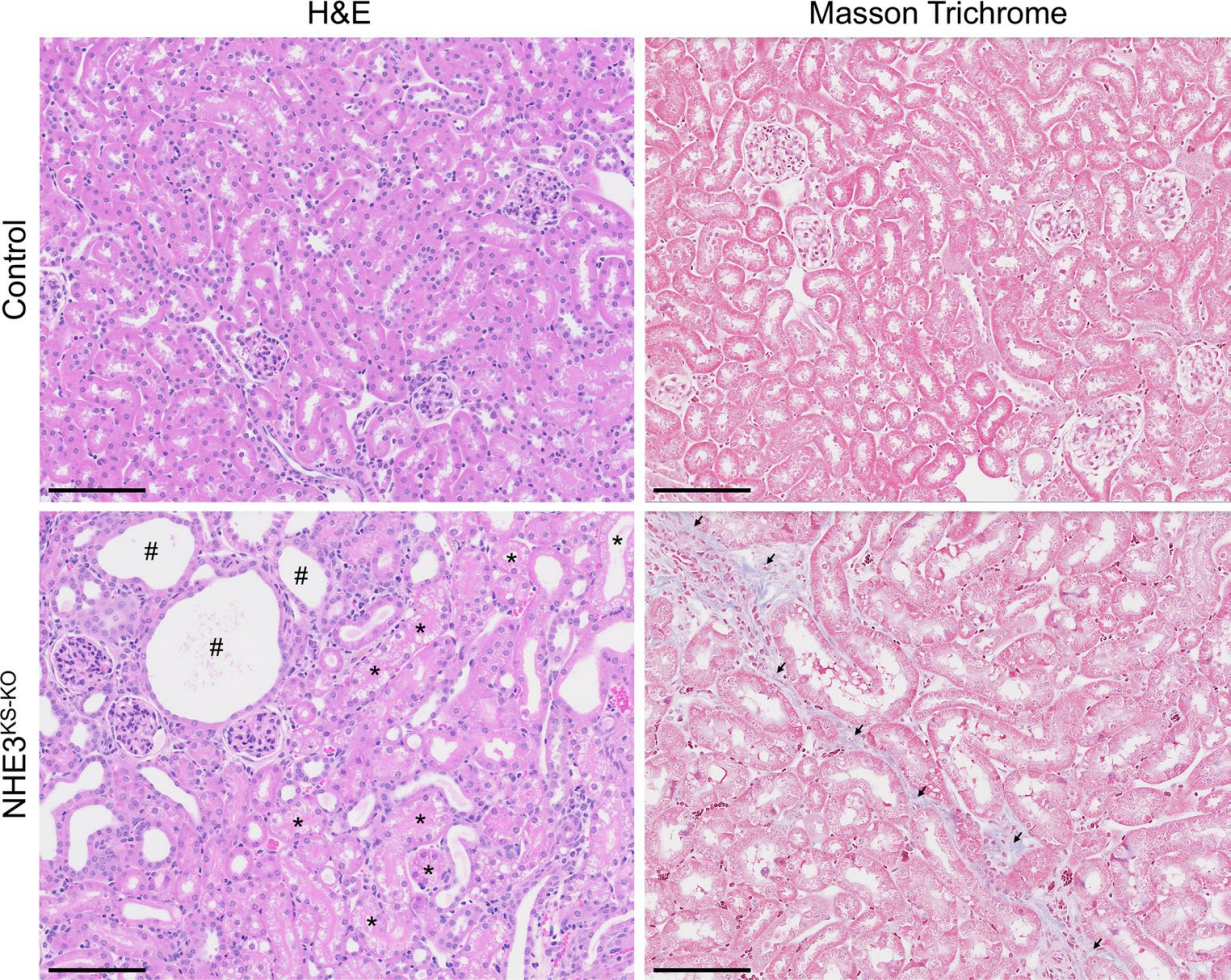
NHE3^KS−KO^ mice show early onset hypokalemic nephropathy. Representative images of H&E and Masson Trichrome staining are shown for control and NHE3^KS−KO^ mice. No histopathological findings were observed in control mice in response to K^+^ deficient diet. In contrast, 3 out of 6 NHE3^KS−KO^ mice showed signs of tubulointerstitial injury, patchy tubular dilation (denoted by #), patchy tubular vacuolization (denoted by *), and focal tubular epithelial cell hyperplasia. Arrows in Masson Trichrome stained slide denote mild interstitial fibrosis. Magnification × 200. Scale bar of 100 μm is shown in each image

Hypokalemia can induce kidney injury, inflammation and fibrosis. Histological analysis of control mice did not show any pathological features or fibrosis (Fig. 8). Of note, three out of the six NHE3^KS−KO^ mice showed histopathological changes: two NHE3^KS−KO^ mice developed grade 1 (< 10%) and one NHE3^KS−KO^ mouse developed grade 2 (10–25%) tubulointerstitial injury. Other features observed were optically clear intracytoplasmic vacuoles with predominantly fine vacuolization of tubular epithelial cells, immune cell infiltration and dilation of tubules (Fig. 8).

## Discussion

K^+^ homeostasis and acid-base regulation are intricately linked and interdependent. Previous studies demonstrated that K^+^ restriction significantly increases renal NHE3 abundance [9, 19, 41]; however, whether this increase is required to develop or sustain metabolic alkalosis through enhanced H^+^ secretion and HCO_3_^-^ reabsorption remained unclear. We hypothesized that renal NHE3 is required for this effect. The present study demonstrates that renal NHE3 mitigates the severity of hypokalemia and metabolic acidosis but does not contribute to the development of metabolic alkalosis. Importantly, these effects occurred independent of changes in GFR, which had been previously proposed as a mechanism to maintain metabolic alkalosis during K^+^ depletion through reduced HCO_3_^-^ filtration [15]. Of note, since all mice on the K^+^-deficient diet developed metabolic acidosis, we limited our ability to test the original hypothesis.

Dietary K^+^ restriction is associated with the development of a urinary concentrating defect [4]. Consistent with this, both control and NHE3^KS-KO^ mice developed nephrogenic diabetes insipidus and polydipsia, with a positive correlation between blood K^+^ concentration and urine osmolality. As expected, urinary K^+^ excretion was nearly completely suppressed, and aldosterone levels were reduced by > 50% in response to dietary K^+^ restriction. Aldosterone regulates the abundance of ROMK and BK channels [47], and the aldosterone-sensitive distal nephron fine-tunes K^+^ homeostasis [37]. The similar abundance of these channels (and localization of ROMK) between genotypes indicates that differential channel expression does not explain the more severe hypokalemia observed in NHE3^KS-KO^ mice. However, because the activity of both ROMK [39] and BK [26] channels is sensitive to intracellular pH, future studies should determine whether channel open probability differs between genotypes. Of note, since there is no need for K^+^ secretion under these conditions, open probability is expected to be low [59]. The data also demonstrate that increases in tubular flow (as indirectly assessed by increases in fluid intake and decreases in urine osmolality) and Na^+^ delivery (as evidenced by increases in urinary Na^+^/creatinine ratios in control mice) to the aldosterone-sensitive distal nephron are offset such that flow-induced and driving force-dependent K^+^ secretion are minimized, allowing K^+^ conservation to predominate.

Suppression of aldosterone during K^+^ restriction cannot explain the hypernatremia that developed in both genotypes. Only control mice increase durinary Na^+^ excretion compared to baseline, suggesting that renal NHE3 is required for the natriuretic response to K^+^ depletion. We hypothesize that volume contraction occurs in this model, analogous to the pathophysiology of lithium-induced nephrogenic diabetes insipidus [8]. Consistent with this hypothesis, we observed significant hemoconcentration at the end of the experimental period, with hematocrit levels increasing from baseline in both control (2.1±0.5%, *P* <0.05) and NHE3^KS-KO^ mice (2.4±0.8%, *P* <0.05). This phenomenon is further supported by clinical observations in humans where hypokalemia – induced by gastric drainage – results in contracted extra cellular volume [30]. Collectively, these findings suggest a physiological hierarchy in which the preservation of K^+^ homeostasis takes precedence over the maintenance of Na^+^ and volume homeostasis.

Although all mice developed hypokalemia as anticipated, they exhibited metabolic acidosis rather than the predicted metabolic alkalosis. A wide range of acid-base responses to K^+^ depletion have been reported, ranging from metabolic alkalosis [9, 12, 32, 41] to metabolic acidosis [10, 11, 38, 54]. The reasons for these varying responses remain unclear but may include: (i) differences in dietary composition (particularly the accompanying anion), (ii) duration of K^+^ restriction, and (iii) species or strain differences. The present data also suggest a respiratory component in the compensatory response, with greater hypercapnia in NHE3^KS−KO^ mice potentially contributing to their more severe acidosis. Further studies utilizing whole-body plethysmography in awake mice would be necessary to fully characterize the ventilatory response.

Renal NH_4_^+^ metabolism and transport are major mechanisms regulating acid-base homeostasis [24, 58], and several studies have established the roles of various enzymes and transporters involved in NH_4_^+^ metabolism and handling during hypokalemia and hyperkalemia [23, 42, 51, 57]. Low K^+^ is a potent stimulus for renal ammoniagenesis [29, 51]. Although the experimental design precluded comparison of mRNA expression to baseline conditions, the data provide evidence that at the end of the experimental period, no genotypic differences existed in mRNA expression of Pepck or Gls, both involved in renal ammoniagenesis [2, 14], or Rhcg, an NH_4_^+^ transporter expressed in the distal tubule/collecting duct which is required for normal ammonia excretion [7]. Glul catalyzes the conversion of ammonium and glutamate to glutamine, thereby decreasing NH_4_^+^ availability for net acid excretion [55]. Whole kidney Glul mRNA expression was significantly lower (~ 60%) in NHE3^KS−KO^ mice compared to control mice. The greater metabolic acidosis in NHE3^KS−KO^ mice may have contributed to lower Glul expression, consistent with prior reports on Glul activity and protein abundance [16]. Since Glul is expressed in both the proximal tubule and type A intercalated cells of the collecting duct, and hypokalemia reduces Glul expression in the proximal tubule while increasing expression in type A intercalated cells, it remains unclear in which segments and/or cell types these differences between NHE3^KS−KO^ and control mice occurred, warranting further investigation. Consistent with the lower urinary NH_4_^+^/creatinine ratios in conjunction with reduced Glul expression in NHE3^KS−KO^ mice, proximal tubule-specific Glul knockout mice show an impaired increase in urinary NH_4_^+^ excretion in response to metabolic acidosis compared to baseline conditions [33]. Together this might imply that proximal tubule NH_4_^+^ secretion is mediated, at least partially (~ 25% lower urinary NH_4_^+^/creatinine ratios in response to dietary K^+^ restriction in NHE3^KS−KO^ versus control mice), by NHE3; however, under baseline conditions NHE3 does not seem to be a rate limiting factor for NH_4_^+^ secretion. Future studies focusing on protein abundances would be helpful to support our mRNA expression data.

In 1937 Schrader et al. [48] described histopathological hallmarks in the kidneys of rats which had been placed on a K^+^ deficient diet (hypokalemic nephropathy). Subsequent studies identified that similar changes exist in humans and rats in response to hypokalemia [40]; these changes include vacuoles in the cortical tubules, tubulointerstitial fibrosis, dilation of tubules and immune cell infiltration. Of note, NaHCO_3_-induced metabolic alkalosis in the presence of hypokalemia significantly attenuated tubulointerstitial injury [53], suggesting that the acid-base disturbance (acidosis versus alkalosis) in response to hypokalemia determines the kinetics and severity of hypokalemic nephropathy. Our data expand this knowledge and suggest that renal NHE3 has a protective effect for the development of hypokalemic nephropathy. Additional studies are needed to determine the relationship between urinary NH_4_^+^, acid-base disturbance, and the development of hypokalemic nephropathy.

In summary, we have demonstrated that while renal NHE3 is not essential for survival and NHE3^KS−KO^ mice can maintain systemic K^+^ homeostasis, its absence necessitates a significant physiological adaptation and predisposes to early onset hypokalemic nephropathy. Although NHE3^KS−KO^ mice exhibited similar qualitative responses compared to control mice, both hypokalemia and metabolic acidosis were significantly exacerbated. Our findings highlight that renal NHE3 serves to attenuate metabolic acidosis during dietary K^+^ restriction and likely contributes to NH_4_^+^ secretion. Given that increased ammoniagenesis typically facilitates K^+^ conservation, the lower NH_4_^+^/creatinine ratio observed in NHE3^KS−KO^ mice may have directly contributed to the observed greater deficit in blood K^+^. Notably, the greater metabolic acidosis in these mice should have provided a potent stimulus for ammoniagenesis; however, since both acidosis and hypokalemia independently drive NH_4_^+^ excretion, the relative contribution of these factors to the NHE3^KS−KO^ phenotype remains to be fully elucidated.

## Supplementary Information

Below is the link to the electronic supplementary material.

Supplementary Material 1 (PDF 455 KB)

## Data Availability

Data will be made available upon reasonable request.

## Abbreviations

BK: big-conductance potassium channel
NHE3: sodium-hydrogen exchanger isoform 3
GFR: glomerular filtration rate
FITC: fluorescein isothiocyanate
ROMK: renal outer medullary potassium channel

## References

[1] Aber GM, Sampson PA, Whitehead TP, Brooke BN (1962) The role of chloride in the correction of alkalosis associated with potassium depletion. Lancet 2:1028–1030. 10.1016/s0140-6736(62)92709-5 14010672 10.1016/s0140-6736(62)92709-5

[2] Alleyne GA, Scullard GH (1969) Renal metabolic response to acid base changes. I. Enzymatic control of ammoniagenesis in the rat. J Clin Invest 48:364–370. 10.1172/JCI105993 4303457 10.1172/JCI105993 PMC322228

[3] Amemiya M, Tabei K, Kusano E, Asano Y, Alpern RJ (1999) Incubation of OKP cells in low-K^+^ media increases NHE3 activity after early decrease in intracellular pH. Am J Physiol 276:C711–716. 10.1152/ajpcell.1999.276.3.C711 10069999 10.1152/ajpcell.1999.276.3.C711

[4] Amlal H, Krane CM, Chen Q, Soleimani M (2000) Early polyuria and urinary concentrating defect in potassium deprivation. Am J Physiol Ren Physiol 279:F655–663. 10.1152/ajprenal.2000.279.4.F655 10.1152/ajprenal.2000.279.4.F655 10997915

[5] Bailey MA, Cantone A, Yan Q, MacGregor GG, Leng Q, Amorim JB, Wang T, Hebert SC, Giebisch G, Malnic G (2006) Maxi-K channels contribute to urinary potassium excretion in the ROMK-deficient mouse model of Type II Bartter’s syndrome and in adaptation to a high-K diet. Kidney Int 70:51–59. 10.1038/sj.ki.5000388 16710355 10.1038/sj.ki.5000388

[6] Bennett CM, Clapp JR, Berliner RW (1967) Micropuncture study of the proximal and distal tubule in the dog. Am J Physiol 213:1254–1262. 10.1152/ajplegacy.1967.213.5.1254 6054873 10.1152/ajplegacy.1967.213.5.1254

[7] Biver S, Belge H, Bourgeois S, Van Vooren P, Nowik M, Scohy S, Houillier P, Szpirer J, Szpirer C, Wagner CA, Devuyst O, Marini AM (2008) A role for Rhesus factor Rhcg in renal ammonium excretion and male fertility. Nature 456:339–343. 10.1038/nature07518 19020613 10.1038/nature07518

[8] Blanco G, Xue J, Thomas L, Dominguez Rieg JA, Sun D, Assmus A, Fenton RA, Rieg T (2025) Lack of renal NHE1 exacerbates lithium-induced nephrogenic diabetes insipidus. Acta Physiol (Oxf) 241:e70029. 10.1111/apha.70029 40123120 10.1111/apha.70029 PMC12924156

[9] Boyd-Shiwarski CR, Weaver CJ, Beacham RT, Shiwarski DJ, Connolly KA, Nkashama LJ, Mutchler SM, Griffiths SE, Knoell SA, Sebastiani RS, Ray EC, Marciszyn AL, Subramanya AR (2020) Effects of extreme potassium stress on blood pressure and renal tubular sodium transport. Am J Physiol Ren Physiol 318:F1341–F1356. 10.1152/ajprenal.00527.2019 10.1152/ajprenal.00527.2019 PMC7311711 32281415

[10] Burnell JM, Dawborn JK (1970) Acid-base parameters in potassium depletion in the dog. Am J Physiol 218:1583–1589. 10.1152/ajplegacy.1970.218.6.1583 5446286 10.1152/ajplegacy.1970.218.6.1583

[11] Burnell JM, Teubner EJ, Simpson DP (1974) Metabolic acidosis accompanying potassium deprivation. Am J Physiol 227:329–333. 10.1152/ajplegacy.1974.227.2.329 4853167 10.1152/ajplegacy.1974.227.2.329

[12] Capasso G, Kinne R, Malnic G, Giebisch G (1986) Renal bicarbonate reabsorption in the rat. I. Effects of hypokalemia and carbonic anhydrase. J Clin Invest 78:1558–1567. 10.1172/JCI112748 3097074 10.1172/JCI112748 PMC423917

[13] Chan YL, Biagi B, Giebisch G (1982) Control mechanisms of bicarbonate transport across the rat proximal convoluted tubule. Am J Physiol 242:F532–543. 10.1152/ajprenal.1982.242.5.F532 6282141 10.1152/ajprenal.1982.242.5.F532

[14] Ching S, Rogoff TM, Gabuzda GJ (1973) Renal ammoniagenesis and tissue glutamine, glutamine synthetase, and glutaminase I levels in potassium-deficient rats. J Lab Clin Med 82:208–214 4146486

[15] Cogan MG, Liu FY (1983) Metabolic alkalosis in the rat. Evidence that reduced glomerular filtration rather than enhanced tubular bicarbonate reabsorption is responsible for maintaining the alkalotic state. J Clin Invest 71:1141–1160. 10.1172/jci110864 6853706 10.1172/JCI110864 PMC436975

[16] Conjard A, Komaty O, Delage H, Boghossian M, Martin M, Ferrier B, Baverel G (2003) Inhibition of glutamine synthetase in the mouse kidney: a novel mechanism of adaptation to metabolic acidosis. J Biol Chem 278:38159–38166. 10.1074/jbc.M302885200 12871952 10.1074/jbc.M302885200

[17] Dominguez Rieg JA, Rieg T (2024) New functions and roles of the Na^+^-H^+^-exchanger NHE3. Pflugers Arch 476:505–516. 10.1007/s00424-024-02938-9 38448727 10.1007/s00424-024-02938-9 PMC12925372

[18] Dominguez Rieg JA, Domenech Acevedo M, Nogueira Coelho J, Stevens M, Thomas L, Rieg T (2025) Distinct roles of ferric carboxymaltose and ferric derisomaltose on phosphate homeostasis in iron deficiency anemia. Eur J Pharm Sci 214:107265. 10.1016/j.ejps.2025.107265 40935025 10.1016/j.ejps.2025.107265 PMC12924155

[19] Elkjaer ML, Kwon TH, Wang W, Nielsen J, Knepper MA, Frokiaer J, Nielsen S (2002) Altered expression of renal NHE3, TSC, BSC-1, and ENaC subunits in potassium-depleted rats. Am J Physiol Ren Physiol 283:F1376–1388. 10.1152/ajprenal.00186.2002 10.1152/ajprenal.00186.2002 12388387

[20] Fenton RA, Poulsen SB, de la Mora Chavez S, Soleimani M, Busslinger M, Dominguez Rieg JA, Rieg T (2015) Caffeine-induced diuresis and natriuresis is independent of renal tubular NHE3. Am J Physiol Ren Physiol 308:F1409–1420. 10.1152/ajprenal.00129.2015 10.1152/ajprenal.00129.2015 PMC4587593 25925253

[21] Fenton RA, Poulsen SB, de la Mora Chavez S, Soleimani M, Dominguez Rieg JA, Rieg T (2017) Renal tubular NHE3 is required in the maintenance of water and sodium chloride homeostasis. Kidney Int 92:397–414. 10.1016/j.kint.2017.02.001 28385297 10.1016/j.kint.2017.02.001 PMC5511580

[22] Gumz ML, Rabinowitz L, Wingo CS (2015) An Integrated View of Potassium Homeostasis. N Engl J Med 373:60–72. 10.1056/NEJMra1313341 26132942 10.1056/NEJMra1313341 PMC5675534

[23] Harris AN, Grimm PR, Lee HW, Delpire E, Fang L, Verlander JW, Welling PA, Weiner ID (2018) Mechanism of hyperkalemia-induced metabolic acidosis. J Am Soc Nephrol 29:1411–1425. 10.1681/ASN.2017111163 29483157 10.1681/ASN.2017111163 PMC5967781

[24] Harris AN, Skankar M, Melanmed M, Batlle D (2023) An update on kidney ammonium transport along the nephron. Adv Kidney Dis Health 30:189–196. 10.1053/j.akdh.2022.12.005 36868733 10.1053/j.akdh.2022.12.005

[25] Hernandez RE, Schambelan M, Cogan MG, Colman J, Morris RC Jr., Sebastian A (1987) Dietary NaCl determines severity of potassium depletion-induced metabolic alkalosis. Kidney Int 31:1356–1367. 10.1038/ki.1987.150 3039234 10.1038/ki.1987.150

[26] Hirano J, Nakamura K, Itazawa S, Sohma Y, Kubota T, Kubokawa M (2002) Modulation of the Ca^2+^-activated large conductance K^+^ channel by intracellular pH in human renal proximal tubule cells. Jpn J Physiol 52:267–276. 10.2170/jjphysiol.52.267 12230803 10.2170/jjphysiol.52.267

[27] Hulter HN, Sebastian A, Sigala JF, Licht JH, Glynn RD, Schambelan M, Biglieri EG (1980) Pathogenesis of renal hyperchloremic acidosis resulting from dietary potassium restriction in the dog: role of aldosterone. Am J Physiol 238:F79–91. 10.1152/ajprenal.1980.238.2.F79 7361893 10.1152/ajprenal.1980.238.2.F79

[28] Jones JW, Sebastian A, Hulter HN, Schambelan M, Sutton JM, Biglieri EG (1982) Systemic and renal acid-base effects of chronic dietary potassium depletion in humans. Kidney Int 21:402–410. 10.1038/ki.1982.36 7070001 10.1038/ki.1982.36

[29] Kamm DE, Strope GL (1973) Glutamine and glutamate metabolism in renal cortex from potassium-depleted rats. Am J Physiol 224:1241–1248. 10.1152/ajplegacy.1973.224.6.1241 4712134 10.1152/ajplegacy.1973.224.6.1241

[30] Kassirer JP, Schwartz WB (1966) The response of normal man to selective depletion of hydrochloric acid. Factors in the genesis of persistent gastric alkalosis. Am J Med 40:10–18. 10.1016/0002-9343(66)90182-3 5901147 10.1016/0002-9343(66)90182-3

[31] Kettritz R, Loffing J (2023) Potassium homeostasis - Physiology and pharmacology in a clinical context. Pharmacol Ther 249:108489. 10.1016/j.pharmthera.2023.108489 37454737 10.1016/j.pharmthera.2023.108489

[32] Kunau RT Jr., Frick A, Rector FC Jr., Seldin DW (1968) Micropuncture study of the proximal tubular factors responsible for the maintenance of alkalosis during potassium deficiency in the rat. Clin Sci 34:223–231 5653684

[33] Lee HW, Osis G, Handlogten ME, Lamers WH, Chaudhry FA, Verlander JW, Weiner ID (2016) Proximal tubule-specific glutamine synthetase deletion alters basal and acidosis-stimulated ammonia metabolism. Am J Physiol Ren Physiol 310:F1229–1242. 10.1152/ajprenal.00547.2015 10.1152/ajprenal.00547.2015 PMC4935770 27009341

[34] Li XC, Soleimani M, Zhu D, Rubera I, Tauc M, Zheng X, Zhang J, Chen X, Zhuo JL (2018) Proximal Tubule-Specific Deletion of the NHE3 (Na^+^/H^+^ Exchanger 3) Promotes the Pressure-Natriuresis Response and Lowers Blood Pressure in Mice. Hypertension 72:1328–1336. 10.1161/HYPERTENSIONAHA.118.10884 30571224 10.1161/HYPERTENSIONAHA.118.10884 PMC6309803

[35] Liu BC, Yang LL, Lu XY, Song X, Li XC, Chen G, Li Y, Yao X, Humphrey DR, Eaton DC, Shen BZ, Ma HP (2015) Lovastatin-Induced Phosphatidylinositol-4-Phosphate 5-Kinase Diffusion from Microvilli Stimulates ROMK Channels. J Am Soc Nephrol 26:1576–1587. 10.1681/ASN.2013121326 25349201 10.1681/ASN.2013121326 PMC4483575

[36] Malnic G, Klose RM, Giebisch G (1964) Micropuncture study of renal potassium excretion in the rat. Am J Physiol 206:674–686. 10.1152/ajplegacy.1964.206.4.674 14166157 10.1152/ajplegacy.1964.206.4.674

[37] McDonough AA, Fenton RA (2022) Potassium homeostasis: sensors, mediators, and targets. Pflugers Arch 474:853–867. 10.1007/s00424-022-02718-3 35727363 10.1007/s00424-022-02718-3 PMC10163916

[38] McKinney TD, Davidson KK (1987) Effect of potassium depletion and protein intake in vivo on renal tubular bicarbonate transport in vitro. Am J Physiol 252:F509–516. 10.1152/ajprenal.1987.252.3.F509 3103470 10.1152/ajprenal.1987.252.3.F509

[39] McNicholas CM, MacGregor GG, Islas LD, Yang Y, Hebert SC, Giebisch G (1998) pH-dependent modulation of the cloned renal K^+^ channel, ROMK. Am J Physiol 275:F972–981. 10.1152/ajprenal.1998.275.6.F972 9843915 10.1152/ajprenal.1998.275.6.F972

[40] Muehrcke RC, Rosen S (1964) Hypokalemic nephropathy in rat and man: a light and electron microscopic study. Lab Invest 13:1359–1373 14226499

[41] Nguyen MT, Yang LE, Fletcher NK, Lee DH, Kocinsky H, Bachmann S, Delpire E, McDonough AA (2012) Effects of K^+^-deficient diets with and without NaCl supplementation on Na^+^, K^+^, and H_2_O transporters’ abundance along the nephron. Am J Physiol Ren Physiol 303:F92–104. 10.1152/ajprenal.00032.2012 10.1152/ajprenal.00032.2012 PMC3431149 22496411

[42] O’Reilly DS (1984) Increased ammoniagenesis and the renal tubular effects of potassium depletion. J Clin Pathol 37:1358–1362. 10.1136/jcp.37.12.1358 6511981 10.1136/jcp.37.12.1358 PMC499025

[43] Onishi A, Fu Y, Darshi M, Crespo-Masip M, Huang W, Song P, Patel R, Kim YC, Nespoux J, Freeman B, Soleimani M, Thomson S, Sharma K, Vallon V (2019) Effect of renal tubule-specific knockdown of the Na^+^/H^+^ exchanger NHE3 in Akita diabetic mice. Am J Physiol Ren Physiol 317:F419–F434. 10.1152/ajprenal.00497.2018 10.1152/ajprenal.00497.2018 PMC6732454 31166707

[44] Polidoro JZ, Luchi WM, Seguro AC, Malnic G, Girardi ACC (2022) Paracrine and endocrine regulation of renal K^+^ secretion. Am J Physiol Ren Physiol 322:F360–F377. 10.1152/ajprenal.00251.2021 10.1152/ajprenal.00251.2021 35073212

[45] Poulsen SB, Murali SK, Thomas L, Assmus A, Rosenbaek LL, Nielsen R, Dimke H, Rieg T, Fenton RA (2025) Genetic deletion of the kidney sodium/proton exchanger-3 (NHE3) does not alter calcium and phosphate balance due to compensatory responses. Kidney Int 107:280–295. 10.1016/j.kint.2024.07.013 39089578 10.1016/j.kint.2024.07.013 PMC12939918

[46] Rector FC Jr., Bloomer HA, Seldin DW (1964) Effect of potassium deficiency on the reabsorption of bicarbonate in the proximal tubule of the rat kidney. J Clin Invest 43:1976–1982. 10.1172/JCI105071 14236221 10.1172/JCI105071 PMC289642

[47] Rieg T, Vallon V, Sausbier M, Sausbier U, Kaissling B, Ruth P, Osswald H (2007) The role of the BK channel in potassium homeostasis and flow-induced renal potassium excretion. Kidney Int 72:566–573. 10.1038/sj.ki.5002369 17579662 10.1038/sj.ki.5002369

[48] Schrader GA, Prickett CO, Salmon WD (1937) Symptomatology and pathology of potassium and magnesium deficiencies in the rat: two plates (Eight Figures). J Nutr 14:85–109. 10.1093/jn/14.1.85

[49] Schultheis PJ, Clarke LL, Meneton P, Miller ML, Soleimani M, Gawenis LR, Riddle TM, Duffy JJ, Doetschman T, Wang T, Giebisch G, Aronson PS, Lorenz JN, Shull GE (1998) Renal and intestinal absorptive defects in mice lacking the NHE3 Na^+^/H^+^ exchanger. Nat Genet 19:282–285. 10.1038/969 9662405 10.1038/969

[50] Soleimani M, Bergman JA, Hosford MA, McKinney TD (1990) Potassium depletion increases luminal Na^+^/H^+^ exchange and basolateral Na^+^:CO_3_^2-^:HCO_3_^-^ cotransport in rat renal cortex. J Clin Invest 86:1076–1083. 10.1172/JCI114810 2170445 10.1172/JCI114810 PMC296834

[51] Tannen RL (1977) Relationship of renal ammonia production and potassium homeostasis. Kidney Int 11:453–465. 10.1038/ki.1977.63 17763 10.1038/ki.1977.63

[52] Thomas L, Dissanayake LV, Tahmasbi M, Staruschenko A, Al-Masri S, Dominguez Rieg JA, Rieg T (2024) Vitamin D_3_ suppresses Npt2c abundance and differentially modulates phosphate and calcium homeostasis in Npt2a knockout mice. Sci Rep 14:16997. 10.1038/s41598-024-67839-4 39043847 10.1038/s41598-024-67839-4 PMC11266651

[53] Tolins JP, Hostetter MK, Hostetter TH (1987) Hypokalemic nephropathy in the rat. Role of ammonia in chronic tubular injury. J Clin Invest 79:1447–1458. 10.1172/JCI112973 3553240 10.1172/JCI112973 PMC424417

[54] van Ypersele C, Dieu JP (1977) Potassium deficiency acidosis in the dog: effect of sodium and potassium balance on renal response to a chronic acid load. Kidney Int 11:335–347. 10.1038/ki.1977.51 19644 10.1038/ki.1977.51

[55] Verlander JW, Chu D, Lee HW, Handlogten ME, Weiner ID (2013) Expression of glutamine synthetase in the mouse kidney: localization in multiple epithelial cell types and differential regulation by hypokalemia. Am J Physiol Ren Physiol 305:F701–713. 10.1152/ajprenal.00030.2013 10.1152/ajprenal.00030.2013 PMC3761201 23804452

[56] Wang T, Yang CL, Abbiati T, Schultheis PJ, Shull GE, Giebisch G, Aronson PS (1999) Mechanism of proximal tubule bicarbonate absorption in NHE3 null mice. Am J Physiol 277:F298–302. 10.1152/ajprenal.1999.277.2.F298 10444585 10.1152/ajprenal.1999.277.2.F298

[57] Weiner ID, Verlander JW (2011) Role of NH_3_ and NH_4_^+^ transporters in renal acid-base transport. Am J Physiol Ren Physiol 300:F11–23. 10.1152/ajprenal.00554.2010 10.1152/ajprenal.00554.2010 PMC3023229 21048022

[58] Weiner ID, Verlander JW (2017) Ammonia Transporters and Their Role in Acid-Base Balance. Physiol Rev 97:465–494. 10.1152/physrev.00011.2016 28151423 10.1152/physrev.00011.2016 PMC5539407

[59] Welling PA, Ho K (2009) A comprehensive guide to the ROMK potassium channel: form and function in health and disease. Am J Physiol Ren Physiol 297:F849–863. 10.1152/ajprenal.00181.2009 10.1152/ajprenal.00181.2009 PMC2775575 19458126

[60] Xue J, Thomas L, Tahmasbi M, Valdez A, Dominguez Rieg JA, Fenton RA, Rieg T (2020) An inducible intestinal epithelial cell-specific NHE3 knockout mouse model mimicking congenital sodium diarrhea. Clin Sci (Lond) 134:941–953. 10.1042/CS20200065 32227118 10.1042/CS20200065 PMC8819665

[61] Xue J, Thomas L, Dominguez Rieg JA, Fenton RA, Rieg T (2022) NHE3 in the thick ascending limb is required for sustained but not acute furosemide-induced urinary acidification. Am J Physiol Ren Physiol 323:F141–F155. 10.1152/ajprenal.00013.2022 10.1152/ajprenal.00013.2022 PMC9306792 35635321

[62] Zemen BG, Lai MH, Whitt JP, Khan Z, Zhao G, Meredith AL (2015) Generation of Kcnma1fl-tdTomato, a conditional deletion of the BK channel alpha subunit in mouse. Physiol Rep 3. 10.14814/phy2.12612 10.14814/phy2.12612 PMC4673641 26537348

