# Supplementary figures and images for: Renal NHE3 is required to limit hypokalemia and metabolic acidosis during dietary potassium deficiency
Source: Pflugers Arch. 2026 Jun 9;478(6):55. doi: 10.1007/s00424-026-03178-9 (PMC13246878; doi:10.1007/s00424-026-03178-9)

**ROMK**

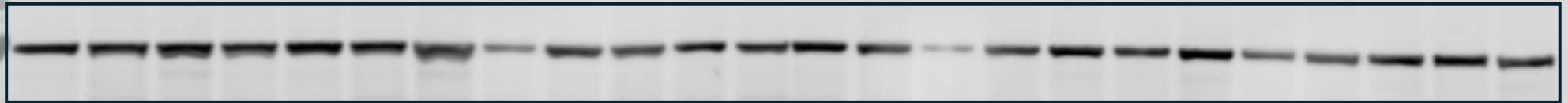

BK $\alpha$

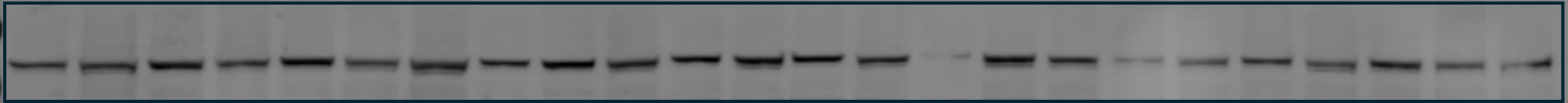

**$\beta$ -actin (BK and ROMK membrane)**

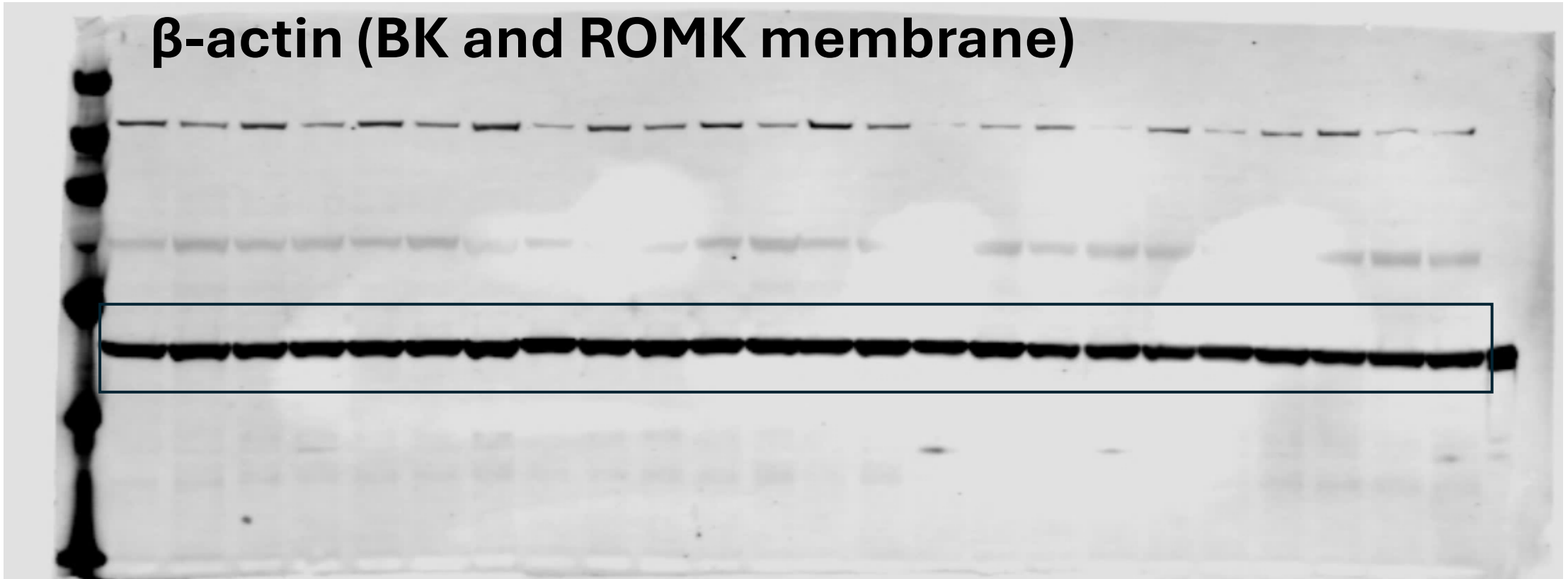

**NHE3**

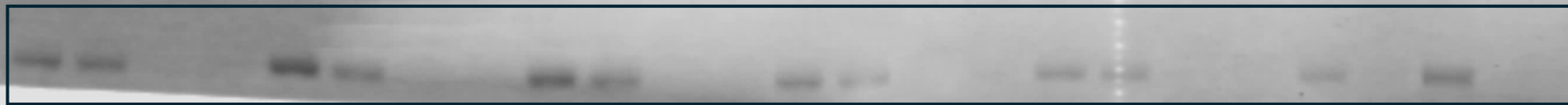

**$\beta$ -actin (NHE3 membrane)**

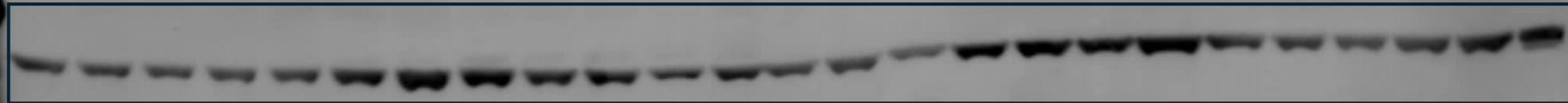

Supplement: Supplementary file 1 — Supplementary Material 1 (PDF 455 KB) [file 424_2026_3178_MOESM1_ESM.pdf]
